# Modeling and Control of a Six Degrees of Freedom Maglev Vibration Isolation System

**DOI:** 10.3390/s19163608

**Published:** 2019-08-19

**Authors:** Qianqian Wu, Ning Cui, Sifang Zhao, Hongbo Zhang, Bilong Liu

**Affiliations:** 1 School of Mechanical and Automotive Engineering, Qingdao University of Technology, Qingdao 266520, China; 2 College of Aerospace and Civil Engineering, Harbin Engineering University, Harbin 150001, China

**Keywords:** magnetic, vibration, dynamic model, control strategy

## Abstract

The environment in space provides favorable conditions for space missions. However, low frequency vibration poses a great challenge to high sensitivity equipment, resulting in performance degradation of sensitive systems. Due to the ever-increasing requirements to protect sensitive payloads, there is a pressing need for micro-vibration suppression. This paper deals with the modeling and control of a maglev vibration isolation system. A high-precision nonlinear dynamic model with six degrees of freedom was derived, which contains the mathematical model of Lorentz actuators and umbilical cables. Regarding the system performance, a double closed-loop control strategy was proposed, and a sliding mode control algorithm was adopted to improve the vibration isolation performance. A simulation program of the system was developed in a MATLAB environment. A vibration isolation performance in the frequency range of 0.01–100 Hz and a tracking performance below 0.01 Hz were obtained. In order to verify the nonlinear dynamic model and the isolation performance, a principle prototype of the maglev isolation system equipped with accelerometers and position sensors was developed for the experiments. By comparing the simulation results and the experiment results, the nonlinear dynamic model of the maglev vibration isolation system was verified and the control strategy of the system was proved to be highly effective.

## 1. Introduction

Disturbances with different acceleration levels in a near-vacuum environment have significant effects on space missions [1,2,3,4]. An assessment indicates that the acceleration level should be below 10^−6^ g to ensure the accuracy of the space activities in a frequency range below 0.01 Hz. The need is somewhat relaxed in a higher frequency range [5,6,7]. Active vibration isolation technology and passive vibration isolation technology are two main methods used to realize vibration isolation. Although a passive vibration isolation technique can provide sufficient attenuation of vibration disturbances in the high frequency range, it is not effective for isolating vibration with low and ultra-low frequencies [8,9]. Magnetostrictive actuators [10], electrodynamics actuators [11], and pneumatic actuators [12] have been successfully applied to the flexible trussed structure and the vehicle suspension system. However, low frequency vibration cannot be isolated by these above actuators. Piezoelectric actuators are widely used in space and ground environments, but it has been proven that the piezoelectric actuator performs poorly in a frequency range below 5 Hz [13,14,15]. By contrast, the Lorentz actuator is ideal for isolating micro-vibration because of its non-contacting and linear characteristics, which have been successfully applied in satellites, spacecraft, and space stations [16,17]. Therefore, a maglev vibration isolation system based on a Lorentz actuator is one of the most promising methods to isolate micro-vibration.

A high precision dynamic model is very important to improve the vibration isolation performance of a maglev vibration isolation system. At present, many researches on dynamic models have been carried out, and some progress has been made, but there are still many problems remaining. Thorsten et al. has presented a model of an active vibration isolation system [18], but only a three degrees-of-freedom (DOF) differential equation of motion was considered. Beadle et al. developed a six DOF rigid body model of the system based on the motion principle of a rigid body [19]. However, the model is not accurate because a local stiffness was used to represent the relationship between the upper platform and the base. In addition, the model is not about a maglev vibration isolation system. A mathematical model of an active vibration isolation platform was constructed on the basis of Lagrange’s mechanics by Zenga [20]. However, the mathematical model of cables was not considered in the system. Hampton et al. built a three DOF translational equation of motion and a three DOF rotational equation of motion with Euler parameters [21]. The model is complicated and cannot represent the real system after multiple linear simplifications. Kim et al. developed a six DOF dynamic model of the g-LIMIT microgravity vibration isolation system [22], which is worth considering. However, simulation and experiment results of the model were not given. Liu et al. established a six DOF nonlinear dynamic model of a vibration isolation system based on Kane’s method [23], but the voice coil actuators were equipped in parallel mechanisms via flexure hinges, which have great influence on the isolation performance. At present, a dynamic model of maglev vibration isolation systems with high precision and high fidelity is still not yet available to ensure the vibration isolation performance.

Classical control methods and modern control methods have been adopted for maglev vibration isolation systems. Traditional PID controllers have been applied to expand control bandwidth [24,25], but only single DOF control simulations were carried out. To improve isolation performance, a PD style double integral acceleration control method was designed by Zhu [6]. However, the control response of a multi-DOF system was not considered. H∞ control algorithms were applied for a cabinet level maglev vibration isolation system [23], but no experiments have been done to verify the control. Self-adaptation control was adopted to compensate for the nonlinearity of magnetic actuators [26,27], but the nonlinear control characteristics were not verified. Optimal feedforward and feedback control methods were applied to the microgravity vibration isolation mount [28], but the control effect was only achieved on a single DOF system model. To sum up, there is the lack of a six DOF dynamic model and experimental verification for the maglev vibration isolation system.

This research aims to develop a high-precision nonlinear dynamic model of a maglev vibration isolation system with six DOF. A double closed-loop control strategy was proposed, and a sliding mode algorithm was studied. A program was developed to obtain the isolation performance and tracking performance of the system. Experiments were conducted to verify the dynamic model and the function of the prototype. This study is helpful to understand the dynamic characteristics and the control behavior of the maglev vibration isolation system.

## 2. Modeling

### 2.1. The Principle of the Maglev Vibration Isolation Platform

**Figure 1:**
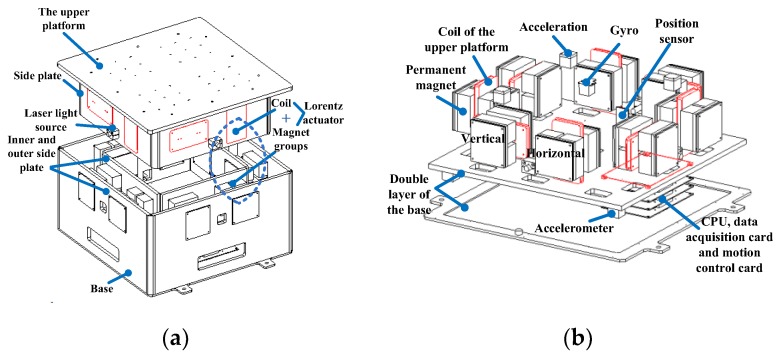
Diagram of the maglev vibration isolation platform: (**a**) General structure diagram; (**b**) Layout of actuators and sensors.

The diagram of the maglev vibration isolation system is shown in Figure 1. The vibration isolation platform was mainly composed of an upper platform and a double-layer base. Eight coils were imbedded in the side plates of the platform. Eight magnet groups were installed between the inner and outer side plates of the base. Lorentz actuators were equipped horizontally and vertically to achieve six DOF control. Three two-dimensional accelerometers were installed on the base and the platform, respectively. Four two-dimensional position sensors and four laser light sources were fixed between the base and the platform [29]. CPU data acquisition cards and motion control cards were all integrated on the base. Currents were distributed to each actuator to generate multi-DOF active control forces to counter the disturbing forces. The maglev vibration isolation platform can be used to eliminate the vibration transmitted to the upper platform in the designed isolation frequency range, as well as maintaining the motion tracking of the base at frequencies lower than the isolation frequency range.

The magnetic field of the Lorentz actuator and the dynamic behavior of the umbilical cables are nonlinear, which has a great influence on the vibration isolation performance. A high-precision dynamic model of the system, containing the precision mathematical model of a Lorentz actuator and the dynamic model of umbilical cables, had to be established firstly for control.

### 2.2. Differential Equation of Motion for the Upper Platform

The upper platform was considered as a rigid body. Six coordinates were used to describe the translational and rotational movement of the upper platform. A state space column matrix was defined as $X={\left[\begin{array}{cc} r & \theta \end{array}\right]}^{T}$, of which $r={\left[x\;y\;z\right]}^{T}$ represents the relative translational movement between the upper platform and the base, and $\theta ={\left[\theta_{x}\;\theta_{y}\;\theta_{z}\right]}^{T}$ represents the rotational movement of the upper platform. Moreover, $\omega ={\left[\begin{array}{ccc} \omega_{x} & \omega_{y} & \omega_{z} \end{array}\right]}^{T}$ was defined as the angular velocity of the platform, and $\alpha ={\left[\begin{array}{ccc} {\dot{\omega }}_{x} & {\dot{\omega }}_{y} & {\dot{\omega }}_{z} \end{array}\right]}^{T}$ was defined as the angular acceleration of the platform. The total force and moment of the platform were defined as $F={\left[\begin{array}{ccc} F_{x} & F_{y} & F_{z} \end{array}\right]}^{T}$ and $M={\left[\begin{array}{ccc} M_{x} & M_{y} & M_{z} \end{array}\right]}^{T}$, respectively.

#### 2.2.1. Establishment of Coordinate Systems and Transformation Matrix

**Figure 2:**
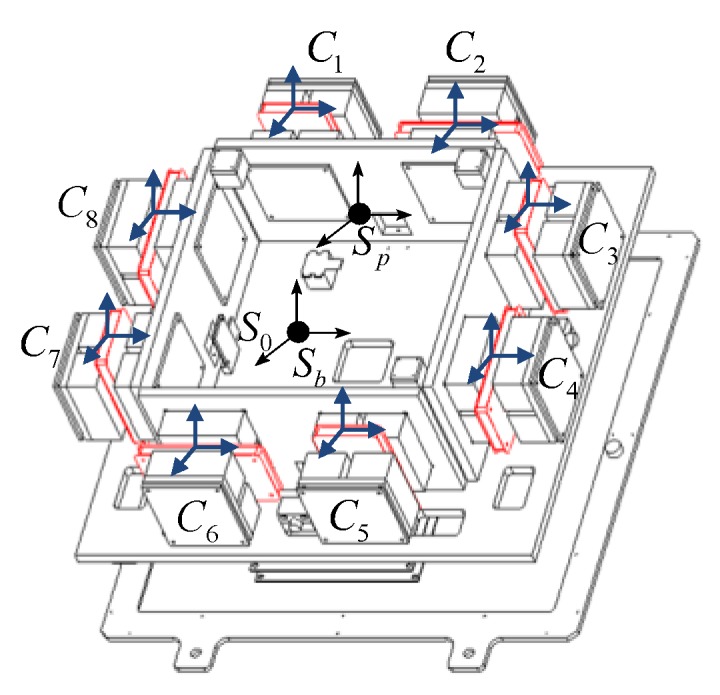
Schematic diagram of coordinate systems.

As shown in Figure 2, the inertial coordinate system was defined as $S_{0}$, with $\Gamma ={\left[\begin{array}{ccc} I & J & K \end{array}\right]}^{T}$ as the orthogonal unit. The platform coordinate system was defined as $S_{p}$ with $\Lambda ={\left[\begin{array}{ccc} i & j & k \end{array}\right]}^{T}$ as the orthogonal unit. The platform coordinate system was set up at the geometry center of the platform. In addition, the base coordinate system was defined as $S_{b}$. At first, the inertial coordinate system overlapped with the base coordinate system. Eight independent actuators with their own coordinate systems (*C*_1_, *C*_2_…*C*_8_) were located at the clockwise azimuths about the *z*-axis. The orthogonal unit was defined as $\Lambda_{a}={\left[\begin{array}{ccc} i_{a} & j_{a} & k_{a} \end{array}\right]}^{T}\left(a=1,\ldots ,8\right)$.

Euler angles of the rotational movement the upper platform were defined as $\theta_{x}$, $\theta_{y}$, and $\theta_{z}$, respectively. A transformation matrix describing the relationship between the platform coordinate system and the inertial coordinate system can be expressed by

(1) $$C=\left[\begin{array}{ccc} c\theta_{y}c\theta_{z} & -c\theta_{y}s\theta_{z} & s\theta_{y}\\ s\theta_{x}s\theta_{y}c\theta_{z}+s\theta_{z}c\theta_{x} & c\theta_{x}c\theta_{z}-s\theta_{x}s\theta_{y}s\theta_{z} & -s\theta_{x}c\theta_{y}\\ s\theta_{z}s\theta_{x}-c\theta_{x}s\theta_{y}c\theta_{z} & c\theta_{x}s\theta_{y}s\theta_{z}+c\theta_{z}s\theta_{x} & c\theta_{x}c\theta_{y} \end{array}\right]$$

where *c* = cos() and *s* = sin().

Based on Euler angles, the relationship between the coordinates of the angular velocity is

(2) $$\left[\begin{array}{c} \omega_{x}\\ \omega_{y}\\ \omega_{z} \end{array}\right]=\left[\begin{array}{ccc} 1 & 0 & s\theta_{y}\\ 0 & c\theta_{x} & -c\theta_{y}s\theta_{x}\\ 0 & s\theta_{x} & c\theta_{x}c\theta_{y} \end{array}\right]\left[\begin{array}{c} {\dot{\theta }}_{x}\\ {\dot{\theta }}_{y}\\ {\dot{\theta }}_{z} \end{array}\right]=D\dot{\theta }$$

where $D=\left[\begin{array}{ccc} 1 & 0 & s\theta_{y}\\ 0 & c\theta_{x} & -c\theta_{y}s\theta_{x}\\ 0 & s\theta_{x} & c\theta_{x}c\theta_{y} \end{array}\right]$.

**Figure 3:**
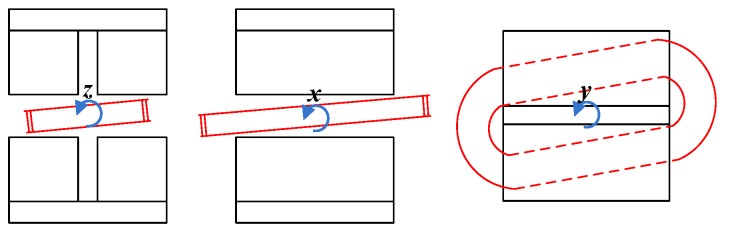
Schematic diagram of attitude change between the coil and the magnet groups.

The schematic diagram of attitude changes between the coil and the magnet groups is shown in Figure 3. When the attitude of the coil changes, the Lorentz force changes too. According to the layout of actuators, the transformation matrix between the actuator coordinate system and the platform coordinate system was defined as $C_{a}$. By analyzing the direction of each Lorentz force under different attitudes, the corresponding transformation matrix can be written as

(3) $$C_{1}=-C_{5}=\left[\begin{array}{ccc} \cos\theta_{x} & 0 & 0\\ 0 & 1 & 0\\ 0 & 0 & 1 \end{array}\right]$$

(4) $$C_{3}=-C_{7}=\left[\begin{array}{ccc} 1 & 0 & 0\\ 0 & \cos\theta_{y} & 0\\ 0 & 0 & 1 \end{array}\right]$$

(5) $$C_{2}=C_{6}=-C_{4}=-C_{8}=\left[\begin{array}{ccc} 1 & 0 & 0\\ 0 & 1 & 0\\ 0 & 0 & \cos\theta_{z} \end{array}\right]$$

According to the relationship between the actuator coordinate system and the platform coordinate system, any vector q in the actuator coordinate system can be transformed to the platform coordinate system by left multiplying the transformation matrix.

(6) $$q^{O}=C_{a}q\;\left(a=1,\ldots ,8\right)$$

where the right superscript ***O*** represents the vector in the platform coordinate system.

Similarly, any vector ***q*** in the actuator coordinate system can be transformed to the initial coordinate system.

(7) $$q^{\Gamma }={\mathrm{CC}}_{a}q\;\left(a=1,\ldots ,8\right)$$

where the right superscript Γ represents the vector in the inertial coordinate system. To simplify the equation, the right superscript Γ was omitted in the paper.

In addition, column matrix was used to express vectors. So, any vector ***q*** can be written as

(8) $$q={\left[\begin{array}{ccc} q_{x} & q_{y} & q_{z} \end{array}\right]}^{T}$$

#### 2.2.2. Definition and Description of Position Vectors

**Figure 4:**
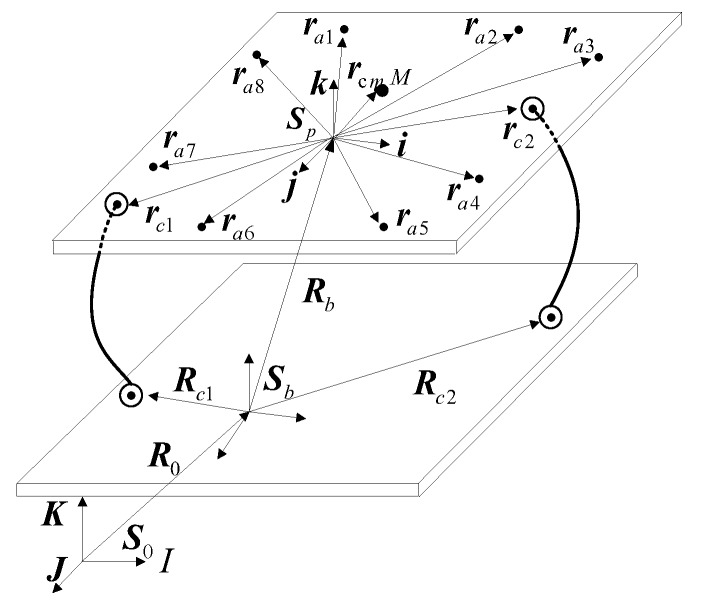
Position vectors of the actuators and installation points of the cables.

Definition and description of displacement vectors are shown in Figure 4. $R_{b}$ is the position vector from the base coordinate system origin to the inertial coordinate system origin. $R_{0}$ and $\theta_{r}$ are the translational movement and rotational movement of the base in the inertial coordinate system. $R_{ci}$ (i = 1,2) are the position vectors from the base coordinate system origin to the installation points of umbilical cables on the base. $r_{ci}$ (i = 1,2) are the position vectors of the installation points in the platform coordinate system. In addition, $r_{a}$ (a = 1,…, 8) are the position vectors from the platform coordinate system origin to the actuator coordinate system origin, respectively. $r_{c}$ is the vector from the platform coordinate system origin to the mass center of the platform. $r_{d}$ is the position vector from the platform coordinate system origin to the location of external force.

#### 2.2.3. Translational Equation of Motion and Rotational Equation of Motion

In the inertial coordinate system, the position vector $r_{\mathrm{cm}}$ of the mass center of the platform can be expressed as

(9) $$r_{\mathrm{cm}}=R_{0}+R_{b}+r+Cr_{c}$$

Then the absolute acceleration of the platform can be solved by differentiation $r_{\mathrm{cm}}$ with time twice.

(10) $$a_{\mathrm{cm}}={\ddot{R}}_{0}+\ddot{r}+C\left(\dot{\omega }\times r_{c}\right)+C\left(\omega \times \left(\omega \times r_{c}\right)\right)$$

According to Newton’s Second Law, the translational equation of motion of the platform can be written as

(11) $$F=m\left({\ddot{R}}_{0}+\ddot{r}+C\left(\dot{\omega }\times r_{c}\right)+C\left(\omega \times \left(\omega \times r_{c}\right)\right)\right)$$

Equation (9) can be transformed as

(12) $$F=m\left({\ddot{R}}_{0}+\ddot{r}-C{\left(r_{c}\right)}^{\sim }\dot{\omega }+C\left({\left({\left(r_{c}\right)}^{\sim }\omega \right)}^{\sim }\omega \right)\right)$$

where the right superscript ${\left(\right)}^{\sim }$ represents the antisymmetric matrix of the vector.

According to the relationship between the angular velocity of the platform ω with Euler angle θ, the translation equation can be expressed by the state space column matrix ***X***.

(13) $$F=mI_{3\times 3}{\ddot{R}}_{0}+m\left[\begin{array}{cc} I_{3\times 3} & -C{\left(r_{c}\right)}^{\sim }D \end{array}\right]\ddot{X}+m\left[\begin{array}{cc} 0_{3\times 3} & C{\left({\left(r_{c}\right)}^{\sim }\omega \right)}^{\sim }D \end{array}\right]\dot{X}$$

According to the Euler equation, the rotation equation can be written as

(14) $$M=CJ_{m}\dot{\omega }+C{\left(\omega \right)}^{\sim }J_{m}\omega$$

Together, the six DOF differential equation of motion of the upper platform is

(15) $$\left[\begin{array}{c} F\\ M \end{array}\right]=\left[\begin{array}{c} mI_{3\times 3}\\ 0_{3\times 3} \end{array}\right]{\ddot{R}}_{0}+\left[\begin{array}{cc} mI_{3\times 3} & -mC{\left(r_{c}\right)}^{\sim }D\\ 0_{3\times 3} & {CJ}_{m}D \end{array}\right]\ddot{X}+\left[\begin{array}{cc} 0_{3\times 3} & mC{\left({\left(r_{c}\right)}^{\sim }\omega \right)}^{\sim }D\\ 0_{3\times 3} & C{\left(\omega \right)}^{\sim }J_{m}D \end{array}\right]\dot{X}$$

#### 2.2.4. Forces and Moments on the Platform

The forces and moments were defined as follows: ***F****_a_* (*a* = 1,2… 8) is the Lorentz force of each actuator; $F_{A}$ is the total force of all the actuators; $F_{c}$ is the disturbing force caused by the deformation of cables; $F_{d}$ is the force acting directly on the upper platform; $M_{A}$ is the total moment of the actuators; $M_{c}$ is the total moment caused by bending deformation of cables; $M_{r}$ is the total moment caused by torsion deformation of cables; and $M_{d}$ is the moment of disturbing force $F_{d}$.

**Figure 5:**
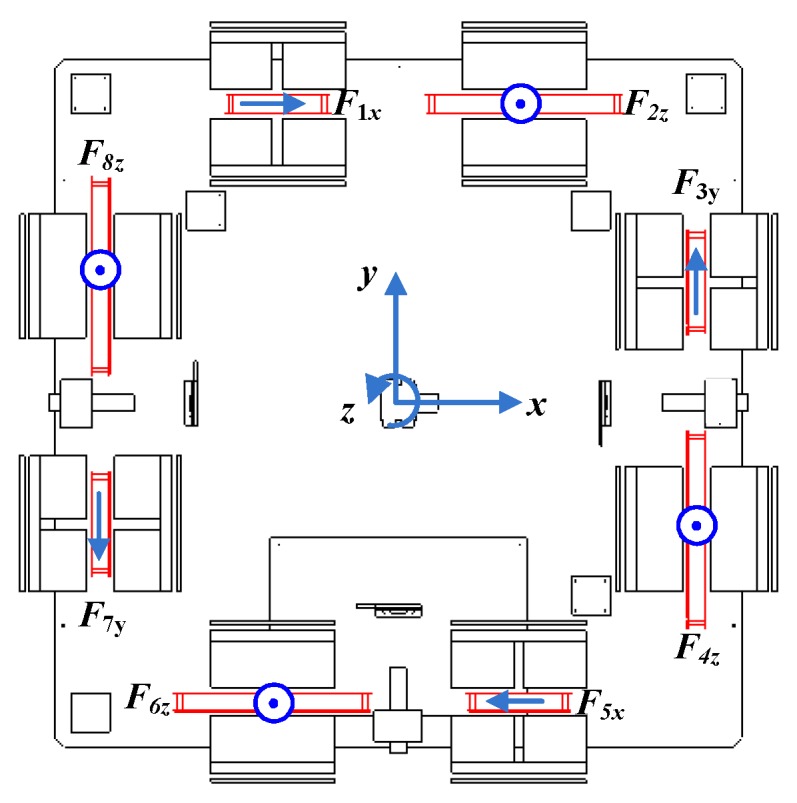
Configuration of Lorentz actuators.

The configuration of Lorentz actuators is displayed in Figure 5. Eight Lorentz forces are produced horizontally and vertically to control six DOF movement. The forces *F*_2_, *F*_4_, *F*_6_, and *F*_8_ are responsible for the vertical force and the moment around the *x*-axis and the *y*-axis. The forces *F*_1_ and *F*_5_ decide the total force along the *x*-axis and moment around the *z*-axis. Finally, the forces *F*_3_ and *F*_7_ decide the total force along the *y*-axis and moment around the *z*-axis.

According to the configuration of the actuators, the Lorentz force vector $f_{0}$ of the actuators is

(16) $$f_{0}={\left[\begin{array}{cccccccc} F_{1x} & F_{2z} & F_{3y} & F_{4z} & F_{5x} & F_{6z} & F_{7y} & F_{8z} \end{array}\right]}^{T}$$

The force $F_{a}$ generated by each actuator can be expressed in the inertial coordinate system.

(17) $$F_{a}={CC}_{a}{\left[\begin{array}{ccc} F_{ax} & F_{ay} & F_{az} \end{array}\right]}^{T}$$

The orientation matrix of each actuator is defined as

(18) $$U={\left[\begin{array}{cccccccc} 1 & & . & . & . & . & & 0\\ 0 & & & & & & & \\ 0 & & & & & & & \\ & 0 & & & & & & \\ & 0 & & & & & & \\ & 1 & & & & & & \\ & & 0 & & & & & \\ & & 1 & & & & & \\ & & 0 & & & & & \\ . & & & 0 & & & & .\\ . & & & 0 & & & & .\\ . & & & 1 & & & & .\\ . & & & & 1 & & & .\\ . & & & & 0 & & & .\\ . & & & & 0 & & & .\\ & & & & & 0 & & \\ & & & & & 0 & & \\ & & & & & 1 & & \\ & & & & & & 0 & \\ & & & & & & 1 & \\ & & & & & & 0 & \\ & & & & & & & 0\\ & & & & & & & 0\\ 0 & & . & . & . & . & & 1 \end{array}\right]}_{24\times 8}$$

Then the sum of eight active control forces $F_{A}$ can be written as

(19) $$F_{A}=\sum_{a=1}^{8}F_{a}=C\left[\begin{array}{ccc} C_{1} & \ldots & C_{8} \end{array}\right]{Uf}_{0}$$

where the position vector of each actuator in the coordinate system of the platform is $r_{Fa}={\left[\begin{array}{ccc} \left(x_{fa}-x_{c}\right) & \left(y_{fa}-y_{c}\right) & \left(z_{fa}-z_{c}\right) \end{array}\right]}^{T}$ (a = 1,2…8). The total moment on the platform from the actuators is

(20) $$M_{A}=\sum_{a=1}^{8}\left({Cr}_{Fa}\times F_{a}\right)=C\left[\begin{array}{ccc} R_{1}C_{1} & \ldots & R_{8}C_{8} \end{array}\right]{Uf}_{0}$$

where $R_{a}={\left(r_{Fa}\right)}^{\sim }$, (*a* = 1,2,3…8).

Most of the system stiffness is dominated by two main power cables. The deformation of the umbilical cables includes bending deformation and torsion deformation. The disturbing forces of the umbilical cables can be obtained approximately by multiplying the equivalent stiffness coefficient with deformation, and by multiplying the equivalent damping coefficient with the derivative of the deformation with respect to time, and then carry on accumulation.

According to the geometry relationship among position vectors, the absolute deformation vectors of umbilical cables $L_{i}$ can be calculated as

(21) $$\begin{array}{ll} L_{i} & =R_{b}+r+Cr_{ci}-R_{ci}-L_{0i}\\ & =\left(R_{b}+r_{ci}-R_{ci}-L_{0i}\right)+r+\left(C-I_{3\times 3}\right)r_{ci}\\ & \approx {\left[\begin{array}{ccc} x & y & z \end{array}\right]}^{T}-{\left(r_{ci}\right)}^{\sim }{\left[\begin{array}{ccc} \theta_{x} & \theta_{y} & \theta_{z} \end{array}\right]}^{T}\\ & =\left[\begin{array}{cc} I_{3\times 3} & -{\left(r_{ci}\right)}^{\sim } \end{array}\right]X \end{array}$$

The force caused by umbilical cables can be written as

(22) $$F_{c}=\sum_{i=1}^{2}\left(K_{ci}\left[\begin{array}{cc} I_{3\times 3} & -{\left(r_{ci}\right)}^{\sim } \end{array}\right]X+C_{ci}\left[\begin{array}{cc} I_{3\times 3} & -{\left(r_{ci}\right)}^{\sim } \end{array}\right]\dot{X}\right)$$

where $K_{ci}$ is the equivalent stiffness matrix of the cables and $C_{ci}$ is the equivalent damping matrix. Then the moment caused by the umbilical cables is

(23) $$\begin{array}{ll} M_{c} & =\sum_{i=1}^{2}\left(r_{fi}\times F_{ci}\right)\\ & =\sum_{i=1}^{2}\left({Cr}_{fi}\times \left(K_{ci}\left[\begin{array}{cc} I_{3\times 3} & -{\left(r_{ci}\right)}^{\sim } \end{array}\right]{X+C}_{ci}\left[\begin{array}{cc} I_{3\times 3} & -{\left(r_{ci}\right)}^{\sim } \end{array}\right]\dot{X}\right)\right)\\ & =\sum_{i=1}^{2}\left({\left({Cr}_{fi}\right)}^{\sim }\left(K_{ci}\left[\begin{array}{cc} I_{3\times 3} & -{\left(r_{ci}\right)}^{\sim } \end{array}\right]{X+C}_{ci}\left[\begin{array}{cc} I_{3\times 3} & -{\left(r_{ci}\right)}^{\sim } \end{array}\right]\dot{X}\right)\right) \end{array}$$

where the rotational stiffness and rotational damping matrices are $K_{ri}$ and $C_{ri}$, respectively. $\theta_{r0}$ is the initial twisting angle, so the relative angle can be written as

(24) $$\theta_{r}=\theta -\theta_{r0}$$

Then torsional moment $M_{r}$ caused by vibration can be written as

(25) $$\begin{array}{ll} M_{r}= & \sum_{i=1}^{2}\left(K_{ri}\left(\theta -\theta_{r0}\right)+C_{ri}\left(\dot{\theta }-{\dot{\theta }}_{r0}\right)\right)\\ = & \sum_{i=1}^{2}\left(K_{ri}\left[\begin{array}{cc} 0_{3\times 3} & I_{3\times 3} \end{array}\right]{X+C}_{ri}\left[\begin{array}{cc} 0_{3\times 3} & I_{3\times 3} \end{array}\right]\dot{X}\right)\\ & -\sum_{i=1}^{2}\left(K_{ri}\theta_{r0}{+C}_{ri}{\dot{\theta }}_{r0}\right) \end{array}$$

where $f_{d}$ is the force exerted directly on the platform. The disturbance force in the inertial coordinate system is

(26) $$F_{d}={Cf}_{d}$$

The directly disturbance moment on the platform is

(27) $$M_{d}=\left(r_{d}-r_{c}\right)\times f_{d}={Cr}_{fd}\times {Cf}_{d}=C{\left(r_{fd}\right)}^{\sim }f_{d}$$

where $r_{fd}$ is the position vector from the acting point of external force on the floating platform to the mass center of the platform.

The total force on the platform can be calculated by

(28) $$\begin{array}{cc} F= & F_{A}-F_{c}+F_{d}\\ = & C\left[\begin{array}{ccc} C_{1} & \ldots & C_{8} \end{array}\right]Uf_{a}+Cf_{d}\\ & -\sum_{i=1}^{2}\left(K_{ci}\left[\begin{array}{cc} I_{3\times 3} & -{\left(r_{ci}\right)}^{\sim } \end{array}\right]X+C_{ci}\left[\begin{array}{cc} I_{3\times 3} & -{\left(r_{ci}\right)}^{\sim } \end{array}\right]\dot{X}\right) \end{array}$$

The total moment on the platform can be obtained by

(29) $$\begin{array}{cc} M= & M_{A}-M_{c}-M_{r}+M_{d}\\ & =C\left[\begin{array}{ccc} R_{1}C_{1} & ... & R_{8}C_{8} \end{array}\right]Uf_{a}+C{\left(r_{fd}\right)}^{\tilde{\;}}f_{d}\\ & -\sum_{i=1}^{2}\left({\left(Cr_{\mathrm{fi}}\right)}^{\tilde{\;}}\left(K_{ci}\left[\begin{array}{cc} I_{3\times 3} & -{\left(r_{ci}\right)}^{\sim } \end{array}\right]X+C_{ci}\left[\begin{array}{cc} I_{3\times 3} & -{\left(r_{ci}\right)}^{\sim } \end{array}\right]\dot{X}\right)\right)\\ & -\sum_{i=1}^{2}\left(K_{ri}\left[\begin{array}{cc} 0_{3\times 3} & I_{3\times 3} \end{array}\right]X+C_{ri}\left[\begin{array}{cc} 0_{3\times 3} & I_{3\times 3} \end{array}\right]\dot{X}\right)+\sum_{i=1}^{2}\left(K_{ri}\theta_{r0}+C_{ri}{\dot{\theta }}_{r0}\right) \end{array}$$

Taking Equations (28) and (29) into Equation (15), the differential equations of motion can be arranged as

(30) $$M_{x}\ddot{X}+C_{x}\dot{X}+K_{x}X=F_{u}+F_{w}$$

where

$$M_{x}=\left[\begin{array}{cc} mI_{3\times 3} & -mC{\left(r_{c}\right)}^{\sim }D\\ 0_{3\times 3} & {CJ}_{m}D \end{array}\right]\;C_{x}=\left[\begin{array}{cc} 0_{3\times 3} & \mathrm{mC}{\left({\left(r_{c}\right)}^{\sim }\omega \right)}^{\sim }D\\ 0_{3\times 3} & C{\left(\omega \right)}^{\sim }J_{m}D \end{array}\right]+\sum_{i=1}^{2}\left[\begin{array}{c} C_{ci}\left[\begin{array}{cc} I_{3\times 3} & -{\left(r_{ci}\right)}^{\sim } \end{array}\right]\\ {\left({Cr}_{fi}\right)}^{\sim }C_{ci}\left[\begin{array}{cc} I_{3\times 3} & -{\left(r_{ci}\right)}^{\sim } \end{array}\right]+\sum_{i=1}^{2}C_{ri}\left[\begin{array}{cc} 0_{3\times 3} & I_{3\times 3} \end{array}\right] \end{array}\right]\;K_{x}=\sum_{i=1}^{2}\left[\begin{array}{c} K_{ci}\left[\begin{array}{cc} I_{3\times 3} & -{\left(r_{ci}\right)}^{\sim } \end{array}\right]\\ {\left(Cr_{\mathrm{fi}}\right)}^{\sim }K_{ci}\left[\begin{array}{cc} I_{3\times 3} & -{\left(r_{ci}\right)}^{\sim } \end{array}\right]+\sum_{i=1}^{2}K_{ri}\left[\begin{array}{cc} 0_{3\times 3} & I_{3\times 3} \end{array}\right] \end{array}\right]$$

$$F_{u}=\left[\begin{array}{c} C\left[\begin{array}{ccc} C_{1} & \ldots & C_{8} \end{array}\right]\\ C\left[\begin{array}{ccc} R_{1}C_{1} & \ldots & R_{8}C_{8} \end{array}\right] \end{array}\right]U_{0}f_{0}$$

$$F_{w}=-\left[\begin{array}{c} mI_{3\times 3}\\ 0_{3\times 3} \end{array}\right]{\ddot{R}}_{0}+\left[\begin{array}{c} C\\ C{\left(r_{fd}\right)}^{\sim } \end{array}\right]f_{d}+\sum_{i=1}^{2}\left[\begin{array}{c} 0_{3\times 3}\\ C_{ri} \end{array}\right]{\dot{\theta }}_{r0}+\sum_{i=1}^{2}\left[\begin{array}{c} 0_{3\times 3}\\ K_{ri} \end{array}\right]\theta_{r0}.$$

## 3. Control Strategy for the Maglev Vibration Isolation System

**Figure 6:**
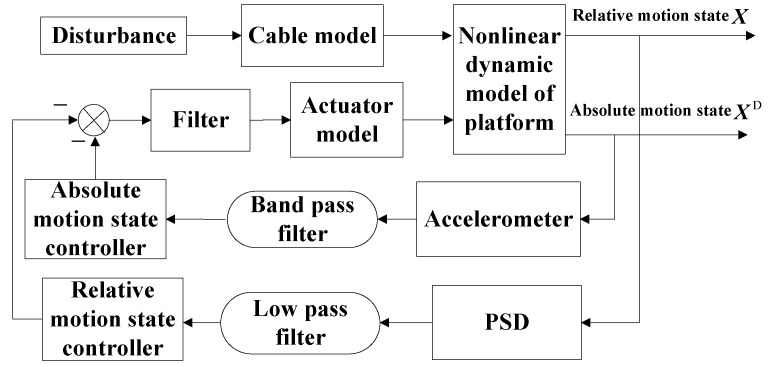
Block diagram of the control system. The absolute displacement error was defined as $e_{a}$, then it can be written as.

Nonlinear dynamics, high response, and a wide frequency band are three typical characteristics of a six DOF maglev vibration isolation platform. The control objective of the system contains two cases. In the isolation frequency range, the goal is to minimize the acceleration level on the upper platform. In the frequency range below the isolation frequency range, the target is to achieve good tracking control between the base and the platform. So, a double closed loop control strategy was proposed, as shown in Figure 6. The inner loop is composed of an absolute movement controller, and the outer loop is composed of a relative movement controller. A low-band filter and a band-pass filter were used to switch the control objective.

(31) $$e_{a}=X_{0}{}^{D}-X^{D}$$

where D is a symbol to represent an absolute variable in the inertial coordinate system Γ and $X_{0}{}^{D}$ is the control target of the absolute displacement of the upper platform. After control, $X_{0}{}^{D}$ should be a constant related to the initial parameters. $X^{D}$ is the actual absolute displacement of the upper platform. Thus, the absolute velocity error is ${\dot{e}}_{a}={\dot{X}}_{0}{}^{D}-{\dot{X}}^{D}$, and the absolute acceleration error is ${\ddot{e}}_{a}={\ddot{X}}_{0}{}^{D}-{\ddot{X}}^{D}$.

A sliding mode surface was defined as follows to achieve vibration isolation:

(32) $$\dot{s}=c_{1}{\dot{e}}_{a}+c_{2}{\ddot{e}}_{a}=c_{1}({\dot{X}}_{0}{}^{D}-{\dot{X}}^{D})+c_{2}({\ddot{X}}_{0}{}^{D}-{\ddot{X}}^{D})$$

where *c*_1_ and *c*_2_ are the coefficients.

According to the stability theory of Lyapunov, a Lyapunov function was defined as

(33) $$V=\frac{1}{2}s^{2}$$

The power approach law was chosen to approach the sliding surface from the initial state. Then it can be obtained as

(34) $$\dot{s}=-k_{1}s-k_{2}{\left|s\right|}^{\alpha }\mathrm{sgn}(s)$$

where *k*_1_ and *k*_2_ are the coefficients. In order to ensure the convergence speed and reduce the oscillation, *k*_1_ can be suitably enlarged and *k*_2_ can be appropriately reduced. The function sgn(s) was defined as follows.

(35) $$\mathrm{sgn}(s)=\{\begin{array}{cc} 1 & s>\beta \\ k_{s}s & \left|s\right|\leq \beta \\ -1 & s<-\beta \end{array}$$

So

(36) $$\dot{V}=s\dot{s}=-k_{1}s^{2}-k_{2}s{\left|s\right|}^{\alpha }\mathrm{sgn}(s)\leq 0$$

where V=0 and s = 0. Then $\dot{V}=s\dot{s}=0$, the control convergence of the system can be ensured.

According to Equations (32) and (34), it can be written as

(37) $$c_{1}({\dot{X}}_{0}{}^{D}-{\dot{X}}^{D})+c_{2}({\ddot{X}}_{0}{}^{D}-{\ddot{X}}^{D})=-k_{1}s-k_{2}{\left|s\right|}^{\alpha }\mathrm{sgn}(s)$$

Then

(38) $${\ddot{X}}^{D}={\ddot{X}}_{0}{}^{D}+\frac{c_{1}({\dot{X}}_{0}{}^{D}-{\dot{X}}^{D})+ks+k_{1}{\left|s\right|}^{\alpha }\mathrm{sgn}(s)}{c_{2}}$$

The acceleration disturbance of the base was defined as ${\ddot{R}}_{d0}$, then ${\ddot{R}}_{d0}={\left[\begin{array}{cc} {\ddot{R}}_{0} & {\ddot{\theta }}_{r} \end{array}\right]}^{T}$. The control force can be obtained by substituting Equation (38) into Equation (30).

(39) $$F_{u}=M_{x}({\ddot{X}}_{0}{}^{D}+\frac{c_{1}({\dot{X}}_{0}{}^{D}-{\dot{X}}^{D})+k_{1}s+k_{2}{\left|s\right|}^{\alpha }\mathrm{sgn}(s)}{c_{2}}-{\ddot{R}}_{d0})+C_{x}\dot{X}+K_{x}X-F_{w}$$

The relative displacement error was defined as $e_{p}$, then it can be written as

(40) $$e_{p}=X_{0}-X$$

where $X_{0}$ is the control target of the relative displacement between the base and the upper platform. After control, $X_{0}$ should be a constant related to the system parameters. Thus, the relative velocity error is ${\dot{e}}_{p}={\dot{X}}_{0}-\dot{X}$ and the relative acceleration error is ${\ddot{e}}_{p}={\ddot{X}}_{0}-\ddot{X}$.

Similarly, a sliding mode surface was defined as Equation (41) to achieve tracking control.

(41) $$s=c_{3}e_{p}+c_{4}{\dot{e}}_{p}$$

where *c*_3_ and *c*_4_ are the coefficients.

The control force for tracking control can be obtained by

(42) $$F_{u}=M_{x}({\ddot{X}}_{0}+\frac{c_{3}({\dot{X}}_{0}-\dot{X})+ks+k_{1}{\left|s\right|}^{\alpha }\mathrm{sgn}(s)}{c_{4}})+C_{x}\dot{X}+K_{x}X-F_{w}.$$

## 4. Simulation and Analysis

A simulation program based on S function was developed in MATLAB/SIMULINK. The isolation performance and tracking performance can be obtained when different disturbances happen. The sliding mode control parameters were adjusted. The larger the coefficient c_1_, the faster it takes to reach the predetermined point, but if the coefficients c_1_ is too large, oscillation would happen. The value of coefficient α has little influence on the response, which can be selected in the range of 0–2. For disturbance that do not vary greatly, the value of β should be smaller, and for disturbance that vary greatly, the value should be bigger.

The physical parameters of the maglev vibration isolation platform used for the simulation are listed in Table 1. In addition, the stiffness matrix and damping matrix of umbilical cables were assumed as

$$K_{ui}=\left[\begin{array}{ccc} 300 & 0 & 0\\ 0 & 300 & 0\\ 0 & 0 & 300 \end{array}\right]\left(N/m\right)$$

$$C_{ui}=\left[\begin{array}{ccc} 24 & 0 & 0\\ 0 & 24 & 0\\ 0 & 0 & 24 \end{array}\right]\left(N\cdot s/m\right)$$

### 4.1. The Principle of the Maglev Vibration Isolation Platform

**Figure 7:**
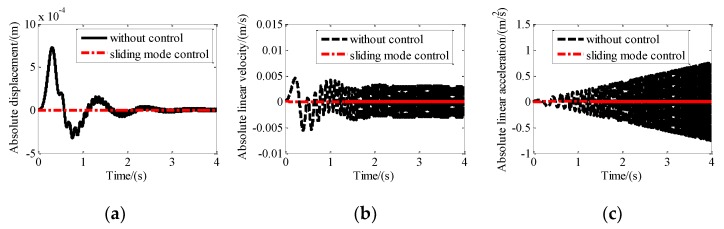
Motion of the platform with and without isolation control along X direction: (**a**) Absolute displacement; (**b**) absolute linear velocity; (**c**) absolute linear acceleration.

A sweep disturbance with an amplitude of 1 mm from 0.01 Hz to 100 Hz was assumed to be exerted on the base of the maglev vibration isolation system. The control parameters are listed as *c*_1_ = 1; *c*_2_ = 0.01; *k* = 100; *m* = 1; α = 1.1; and β = 0.1. If we take the response of the upper platform along *x*-axis as an example, the change of absolute variables with and without sliding mode control are shown in Figure 7. With the increase of frequency, the amplitude of acceleration without control increases gradually, and the switching period of velocity direction decreases. So, the amplitude of vibration displacement and velocity without control does not increase with the increase of frequency, while the amplitude of acceleration without control increases with the increase of frequency. Moreover, it can be seen that the absolute displacement, absolute velocity, and absolute acceleration of the upper platform with sliding mode control can be suppressed quickly. That means the designed maglev vibration isolation can achieve isolation control in the range of 0.01–100 Hz.

### 4.2. Vibration Isolation Control Simulation Under Step Disturbance

**Figure 8:**
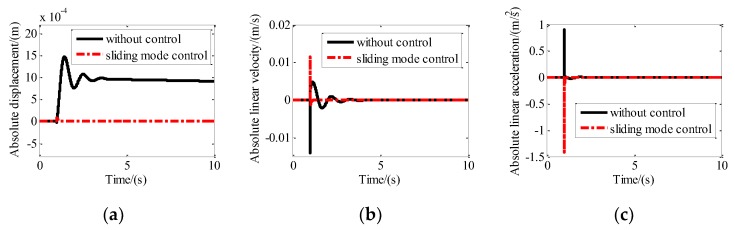
Motion of the platform with and without sliding mode control along the x direction: (**a**) Absolute displacement; (**b**) absolute linear velocity; (**c**) absolute linear acceleration.

Step excitation with an amplitude of 1 mm was assumed to be exerted on the base of the maglev vibration isolation platform along the *X*-axis, *Y*-axis, and *Z*-axis, simultaneously. The control parameters used for the simulation are *c*_1_ = 1; *c*_2_ = 0.01; *k* = 20; *m* = 1; α = 1.1; and β = 0.001. The absolute displacement, absolute velocity, and absolute acceleration of the upper platform with and without sliding mode control along the X direction are shown in Figure 8. It can be seen that the absolute displacement approaches 1 mm after about five seconds. The absolute velocity and the absolute acceleration approach zero after about five seconds. After control, the absolute displacement, absolute velocity, and absolute acceleration of the platform approach zero quickly.

### 4.3. Tracking Control Simulation Under Sinusodial Disturbance

**Figure 9:**
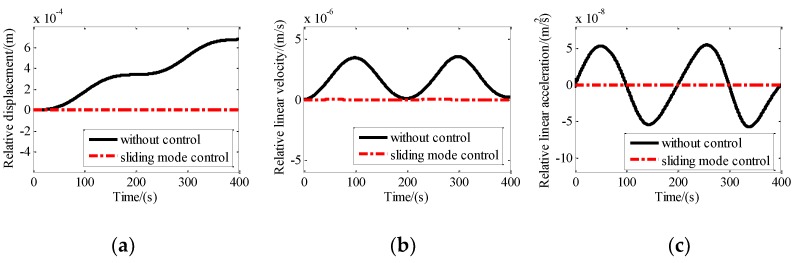
Motion of the platform with and without tracking control along the X direction: (**a**) Relative displacement; (**b**) relative linear velocity; (**c**) relative linear acceleration.

To obtain the tracking performance of the maglev vibration isolation platform, sine disturbance with an amplitude of 2 mm and a frequency of 0.005 Hz along the *X*-axis was exerted on the base. The control parameters are adjusted as: *c*_1_ = 1; *c*_2_ = 0.01; *k* = 0.05; *m* = 1; α = 1.1; and β = 0.01. The relative displacement, relative velocity, and relative acceleration between the base and the platform are shown in Figure 9. It indicates that the relative motion reached zero quickly after control. The comparison between the disturbance and the absolute motion of the platform in Figure 9 shows that the motion of the platform is consistent with the disturbance. That means the maglev vibration isolation platform has excellent tracking performance under the sliding mode control.

## 5. Experiments

**Figure 10:**
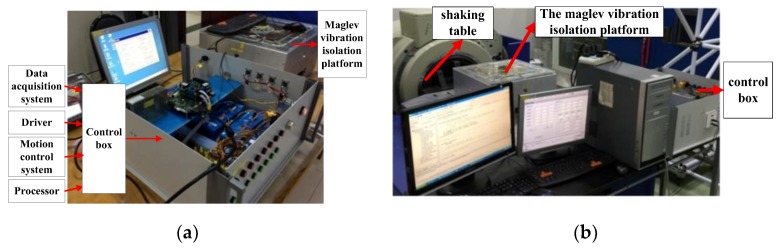
Experimental setup: (**a**) Prototype and control box; (**b**) vibration isolation experiment setup.

A six DOF maglev vibration isolation platform prototype was manufactured, and the test system was built as shown in Figure 10. Accelerometers (Silicon Design Model 2422), two-dimensional PSDs (DL400-7-PCBA) and light sources (LDM635-5LT) were equipped on the prototype. The base of the maglev vibration isolation prototype was fixed on a shaking table. DC components were added into four vertical Lorentz actuators to develop forces compensating the weight of the upper platform. According to the proposed control strategy and control algorithm, experiments were conducted to verify the dynamic and the isolation performance.

### 5.1. Verification of the Dynamic Model of the Maglev Vibration Isolation Platform

**Figure 11:**
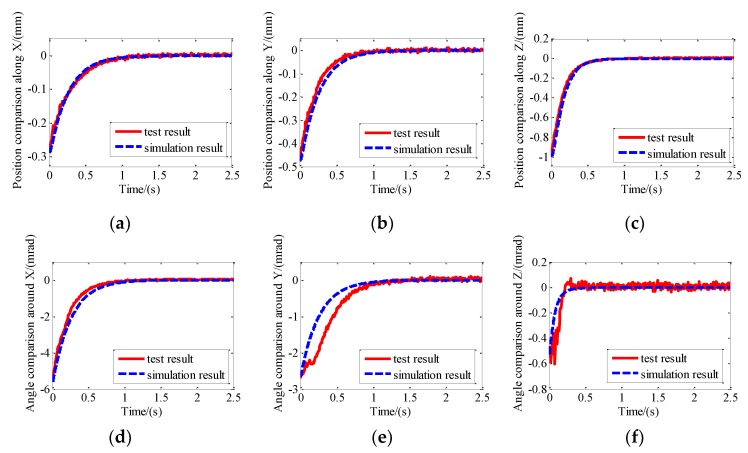
Comparison between simulation results and test results: (**a**) Position comparison along X; (**b**) position comparison along Y; (**c**) position comparison along Z; (**d**) angle comparison around X; (**e**) angle comparison around Y; (**f**) angle comparison around Z.

The physical zero of the photosensitive surface of the position sensor was set as the target zero of the system. The platform moved from the initial position and attitude to the target position and attitude under control. Then, extracting the test results of the PSDs and the initial position and attitude could be obtained. The initial configurations of the system along and around X, Y, and Z were −0.288 mm, −0.478 mm, −1.00 mm, −5.5 mrad, −2.7 mrad, and −0.56 mrad, respectively. Then the initial state of the simulation program was set according to the tested data above. The six DOF relative motion of the maglev vibration isolation platform was obtained under the same controller and target conditions. Comparison between simulation results and test results are shown in Figure 11. The relative motions obtained by the simulation program based on the dynamic model are consistent with the test results, which indicate that the proposed six DOF nonlinear dynamic model of the maglev vibration isolation platform is correct.

### 5.2. Vibration Isolation of the Maglev Vibration Isolation Platform

**Figure 12:**
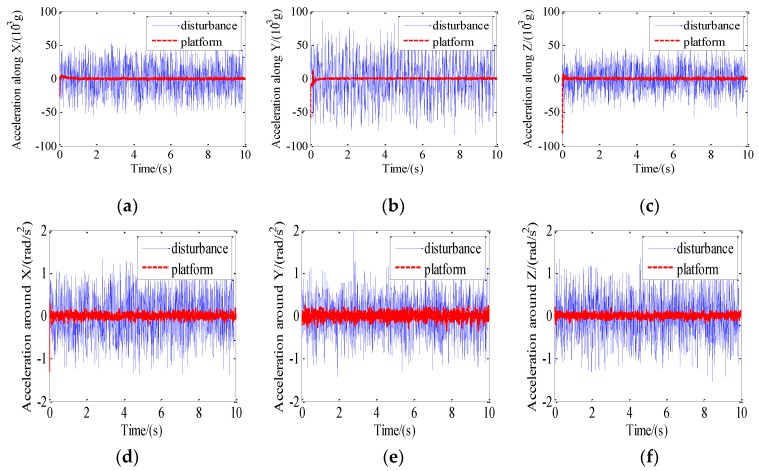
Comparison of the acceleration response of the platform with disturbance: (**a**) Acceleration along X; (**b**) acceleration along Y; (**c**) acceleration along Z; (**d**) acceleration around X; (**e**) acceleration around Y; (**f**) acceleration around Z.

The shaking table was set to generate sinusoidal vibration along the horizontal direction. Then the base would move together with the shaking table. Accelerometers were adopted to measure the acceleration of the base and the platform. The acceleration on the floating platform was consistent with that on the base without control. The control parameters used for the experiments were adjusted as: c_1_ = 2; c_2_ = 0.01; k = 300; m = 1; α = 1.1; and β = 0.05. The comparison of the acceleration response of the platform with disturbance are shown in Figure 12.

The peak-peak acceleration of disturbance and the peak-peak acceleration of the platform after control at the same time interval was compared and the isolation performance is illustrated in Table 2. It can be seen that the percentage of vibration isolation is more than 80% in six DOF.

**Figure 13:**
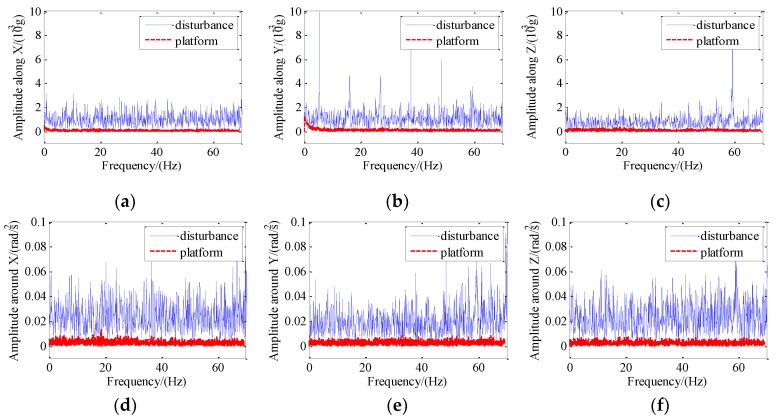
Spectrum analysis of the acceleration of the platform and disturbance: (**a**) Amplitude along X; (**b**) amplitude along Y; (**c**) amplitude along Z; (**d**) amplitude around X; (**e**) amplitude around Y; (**f**) amplitude around Z.

Acceleration frequency spectrum analysis of the platform and disturbance after control is shown in Figure 13. It can be seen that disturbance amplitudes in the frequency range of 0–70 Hz were all suppressed after control.

**Figure 14:**
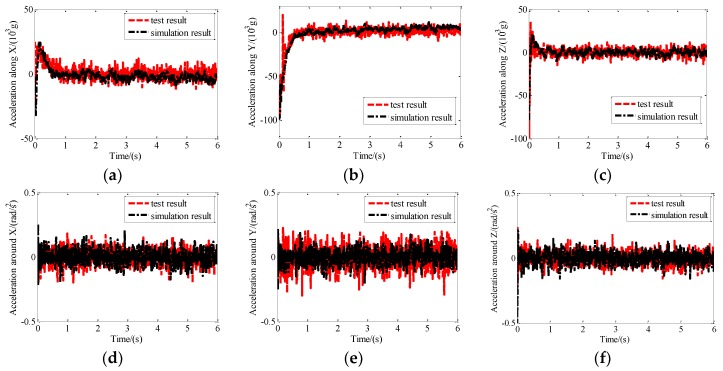
Comparison between simulation results and test results: (**a**) Acceleration along X; (**b**) acceleration along Y; (**c**) acceleration along Z; (**d**) acceleration around X; (**e**) acceleration around Y; (**f**) acceleration around Z.

The measured disturbance was added into the simulation program as input disturbing parameters, and simulation results of control were obtained according to the simulation program of the proposed maglev vibration isolation system. Comparison between the simulation results and the test results are shown in Figure 14. The results expressed that the response trend of acceleration based on the simulation model is consistent with that of the actual system, which further verifies the proposed dynamic model of the maglev vibration isolation platform.

## 6. Conclusions

A maglev vibration isolation platform with six DOF was studied in the paper. A high-precision nonlinear dynamic model of the system was established by introducing the mathematical model of the Lorentz actuator and umbilical cables. In order to achieve micro-vibration isolation of the system and avoid collision between the base and the platform, a double closed loop control strategy was proposed. A sliding mode control algorithm was applied to achieve isolation control in the isolation frequency range and tracking control in the frequency range below the isolation frequency range. A simulation program based on the nonlinear dynamic model and the control algorithm was developed. Good isolation performance for swap disturbance from 0.01 Hz to 100 Hz and step disturbance and good tracking performance have been proven for sine disturbance. A prototype of the maglev isolation system was manufactured, and a test system was established for experiments. The nonlinear dynamic model was verified by comparing the simulation results and the test results about the six DOF motion response of the maglev vibration isolation system. In addition, the vibration isolation performance in both the time domain and the frequency domain was tested. The percentage of vibration isolation is more than 80% in six DOF, and the system can effectively suppress the disturbance within the frequency band of 0–70 Hz. Moreover, the dynamic model was further verified by comparing the absolute acceleration between the test results and the simulation results.

## Figures and Tables

**Table 1:** Physical parameters of the maglev vibration isolation system platform.

Description	Value
*m*/Kg	16
*r_c_*/mm	[10;10;2]
*r_u_*_1_/mm	[−133.8;−204.16;38.09]
*r_u_*_2_/mm	[133.8;204.16;38.09]
*r_d_*/mm	[51.48;−49.45;59.31]
*r_F_*_1*M*_/mm	[−77.4;191.29;32.91]
*r_F_*_2*M*_/mm	[80.2;192.29;32.91]
Moment of the inertia tensor *J*/kg mm^2^	[3.81e5 −571.84 −659.74;
	−571.84 3.81e5 −855.16;
	−659.74 −855.16 6.89e5]

**Table 2:** Vibration isolation ratio of the maglev vibration isolation system.

Direction	Peak-Peak Acceleration of Disturbance	Peak-Peak Acceleration of the Platform	Vibration Isolation Ratio
Along X (mg)	108.16	5.49	94.92%
Along Y (mg)	159.29	5.76	96.38%
Along Z (mg)	113.37	5.12	95.48%
Around X (rad/s2)	2.684	0.385	85.66%
Around Y(rad/s2)	3.051	0.412	86.5%
Around Z(rad/s2)	2.906	0.368	87.34%

## References

[1] Jia T., Chen S., Peng J., Yong W. (2017). Active disturbance rejection control for microgravity active vibration isolation system in space station. Inf. Control.

[2] Sinha A., Kao C.K., Grodsinsky C. (1990). A new approach to controller design for microgravity isolation systems. Acta Astronaut..

[3] Wu Q., Yue H., Liu R., Zhang X., Ding L., Liang T., Deng Z. (2015). Measurement model and precision analysis of accelerometers for maglev vibration isolation platforms. Sensors.

[4] Bushnell G.S., Fialho I.J., Allen J.L., Quraishi N. (2003). Flight performance of the International Space Station active rack isolation system. J. Acoust. Soc. Am..

[5] Grodsinsky C.M., Whorton M.S. (2000). Survey of active vibration isolation systems for microgravity applications. J. Spacecr. Rocket..

[6] Zhu W.H., Tryggvason B., Piedboeuf J.C. (2006). On active acceleration control of vibration isolation systems. Control Eng. Pract..

[7] Wu Q., Liu B., Cui N., Yue H., Liu R. (2019). Dynamic analysis of umbilical cables for maglev vibration isolation systems. AIAA J..

[8] Hong J., Park K. (2010). Design and control of six degree-of-freedom active vibration isolation table. Rev. Sci. Instrum..

[9] Bock T., Jousten K. (2006). Offset scatter reduction of spinning rotor gauges by vibration isolation. Vacuum.

[10] Zhang T., Yang B.T., Li H.G., Meng G. (2013). Dynamic modeling and adaptive vibration control study for giant magnetostrictive actuators. Sens. Actuators A Phys..

[11] Berardi U. (2013). Modelling and testing of a dielectic electro-active polymer (DEAP) actuator for active vibration control. J. Mech. Sci. Technol..

[12] Chen H.Y., Liang J.W. (2017). Adaptive wavelet neural network controller for active suppression control of a diaphragm-type pneumatic vibration isolator. Int. J. Control Autom..

[13] Preumont A., Horodinca M., Romanescu I., Marneffe B.D., Avraam M., Deraemaeker A. (2007). A si*x*-axis single-stage active vibration isolator based on Stewart platform. J. Sound Vib..

[14] Ying W., Yu K., Jian J., Zhao R. (2015). Dynamic modeling and robust nonlinear control of a six-DOF active micro-vibration isolation manipulator with parameter uncertainties. Mech. Mach. Theory.

[15] Wang C., Xie X., Chen Y., Zhang Z. (2016). Investigation on active vibration isolation of a Stewart platform with piezoelectric actuators. J. Sound Vib..

[16] Zhu T., Cazzolato B., Robertson W.S.P., Zander A. (2015). Vibration isolation using six degree-of-freedom quasi-zero stiffness magnetic levitation. J. Sound Vib..

[17] Kim M., Kim H., Gweon D. (2012). Design and optimization of voice coil actuator for six degree of freedom active vibration isolation system using Halbach magnet array. Rev. Sci. Instrum..

[18] Muller T., Hurlebaus S., Stobener U., Gaul L. Modeling and control of an active vibration isolation. Proceedings of the International Modal Analysis Conference IMAC.

[19] Kerber F., Hurlebaus S., Beadle B.M., Stöbener U. (2007). Control concepts for an active vibration isolation system. Mech. Syst. Signal Process..

[20] Zenga A.L.Y. (2005). Iterative SISO feedback design for an active vibration isolation system. Traineesh. Rep..

[21] Hampton R., Tryggvason B., Decarufel J., Townsend M.A., Wagar W.O. (1997). The Microgravity Vibration Isolation Mount: A Dynamic Model for Optimal Controller Design.

[22] Kim Y.K., Whorton M.S. (2001). Equations of Motion for the g-LIMIT Microgravity Vibration Isolation System.

[23] Liu J., Li Y., Zhang Y., Gao Q., Zuo B. (2014). Dynamics and control of a parallel mechanism for active vibration isolation in space station. Nonlinear Dynam..

[24] Fenn R.C., Downer J.R., Gondhalekar V., Johnson B.G. (1990). An active magnetic suspension for space-based micro-gravity vibration isolation. Act. Noise Vib. Control.

[25] Hu Y., Chen C., Wu H., Song C. (2018). Study on structural optimization design and cascade PID control of maglev actuator for active vibration isolation system. J. Vib. Control.

[26] Li Y., He L., Shuai C.G. (2013). Nonlinearity of maglev actuator and adaptive vibration control using improved FxLMS algorithm. Appl. Mech. Mater..

[27] Yang B.J., Calise A., Craig J., Whorton M. (2013). Adaptive Control for a Microgravity Vibration Isolation System.

[28] Hampton R.D., Knospe C., Grodsinsky C., Allaire P.E., Lewis D.W. (1992). Microgravity Vibration Isolation: Optimal Preview and Feedback Control.

[29] Xie L., Qiu Z., Zhang X. (2019). Development of a 3-PRR precision tracking system with full closed-loop measurement and control. Sensors.

